# Relationship of the vascular territory affected by delayed cerebral ischemia and the location of the ruptured aneurysm in patients with aneurysmal subarachnoid hemorrhage

**DOI:** 10.1007/s10143-021-01522-4

**Published:** 2021-03-29

**Authors:** Helene Hurth, Jochen Steiner, Ulrich Birkenhauer, Constantin Roder, Till-Karsten Hauser, Ulrike Ernemann, Marcos Tatagiba, Florian Heinrich Ebner

**Affiliations:** 1 Department of Neurosurgery, University Hospital Tuebingen, Hoppe-Seyler-Straße 3, 72076 Tuebingen, Germany; 2 Department of Anesthesiology and Intensive Care Medicine, University Hospital Tuebingen, Tuebingen, Germany; 3 Department of Neuroradiology, University Hospital Tuebingen, Tuebingen, Germany; 4 Department of Neurosurgery, Alfried-Krupp Hospital, Essen, Germany

**Keywords:** Aneurysmal subarachnoid hemorrhage, Delayed cerebral ischemia, Vascular territory, Location, Neuromonitoring

## Abstract

**Objective:**

To determine the area most at risk of delayed cerebral ischemia (DCI) in relation to the location of the ruptured aneurysm in patients with aneurysmal subarachnoid hemorrhage (aSAH) and, therefore, help to choose the site for focal multimodal neuromonitoring.

**Methods:**

We retrospectively analyzed angiographic findings, CCT scans, and patient charts of patients who were admitted with aSAH to our neurosurgical intensive care unit between 2009 and 2017. DCI was defined as infarction on CCT 2–6 weeks after aSAH.

**Results:**

DCI occurred in 17.9% out of 357 included patients. A DCI occurring in the vascular territory of the artery carrying the ruptured aneurysm was found in 81.0% of patients with anterior circulation aneurysms but only in 16.7% with posterior circulation aneurysms (Fisher’s exact, *p*=0.003). The vascular territory most frequently showing a DCI was the ipsilateral MCA territory (86.7%) in ICA aneurysms, the contra- (71.4%) and the ipsilateral (64.3%) ACA territory in ACA aneurysms, the right (93.8%) and the left (81.3%) ACA territory in AcomA aneurysms, and the ipsilateral MCA territory in MCA aneurysms (69.2%) as well as in VA/PICA/SCA aneurysms (100.0%). DCI after the rupture of a BA aneurysm occurred with 33.3% in 6 out of 8 vascular territories, respectively. DCI of multiple vascular territories occurred in 100.0% of BA aneurysms, 87.5% of AcomA aneurysms, 71.4% of ACA aneurysms, 40.0% of ICA aneurysms, 38.5% of MCA aneurysms, and 33.3% of VA/PICA/SCA aneurysms.

**Discussion:**

Few studies exist that could determine the area most at risk of a DCI after an aSAH. Our data could identify the territory most at risk for DCI with a probability of > 60% except for BA aneurysms, which showed DCI in various areas and patients suffering from multiple DCIs. Either the ipsilateral ACA or MCA were affected by the DCI in about 80% of ACA and more than 90% of AcomA, ICA, MCA, and VA/PICA/SCA aneurysms. Therefore, local intraparenchymal neuromonitoring in the ACA/MCA watershed area might detect the vast majority of DCIs for all aneurysm locations, except for BA aneurysms. In ACA and AcomA aneurysms, bilateral DCI of the ACA territory was common, and bilateral probe positioning might be considered for monitoring high-risk patients. Non-focal monitoring methods might be preferably used after BA aneurysm rupture.

## Introduction

Despite highly specialized treatment and optimized management at neurointensive care units, aneurysmal subarachnoid hemorrhage (aSAH) as the main cause of stroke in young patients is still accompanied by a broad spectrum of possible complications that lead to a poor outcome [2, 6, 10]. One major risk factor for a poor outcome in patients who survived the initial ictus is the development of a delayed cerebral ischemia (DCI). DCI is the result of a multicausal and still incompletely understood mechanism including cerebral vasospasm (VS), spreading cortical depolarization, inflammatory, and microthrombotic processes that occur in approximately 30% of patients[1, 4, 14]. Treatment of DCI consists of the systemic application of nimodipine and the maintenance of moderate hypertension after treatment of the aneurysm [2, 9].

Since DCI usually occurs within the first 2 weeks after the bleeding event, neuroradiologic screening for VS and multimodal neuromonitoring are helpful tools, especially in patients who are not eligible for neurologic examination due to a poor neurological condition or the prolonged necessity for intubation and sedation [2, 12, 15, 16]. Intraparenchymal probes for continuous measurement of cerebral tissue oxygen saturation or interstitial tissue metabolism are commonly used for early diagnosis of an impaired brain perfusion [17]. Although these methods have been shown to be highly sensitive to changes in local brain perfusion, the main limitation is the focal nature of the measurement. Therefore, the location of the probe is essential to obtain the desired benefit. It has been recommended to place microdialysis catheters into the ACA/MCA watershed area on the side of the thicker subarachnoid blood clot or the non-dominant hemisphere [7, 21]. However, systematic investigations determining the area most at risk for the development of a DCI are rare [13, 21].

The aim of this study was to identify the area most frequently affected by a DCI-related infarction in a large patient cohort requiring prolonged NICU treatment and to evaluate the relationship between the location of the ruptured aneurysm and the vascular territories most at risk in these patients.

## Methods

Over an 8-year period from 2009 to 2017, all patients who were treated at the neurosurgical intensive care unit of our tertiary care university hospital due to aSAH were retrospectively analyzed. The inclusion criterion was the existence of at least one CCT scan 24 to 48 h after treatment of the aneurysm as well as another scan a minimum of 14 days after the bleeding event. Patients who had cerebral infarctions due to causes other than DCI (i.e., increased intracranial pressure, complications of surgical or endovascular treatment) or who died within 14 days after the bleeding event were excluded. DCI was defined radiologically as an infarction visible on CCT 2 to 6 weeks after the aSAH as suggested by Vergouwen et al. [22]. Clinical DCI was not included in this analysis.

CCT scans between 2 and 6 weeks after the aSAH were evaluated for the presence of ischemia that was not visible on the CCT 24 to 48 h after aneurysm treatment and that was not attributable to a cause other than DCI. In addition, the presence of severe angiographic VS was assessed. Routine daily transcranial Doppler sonography examinations were performed from day 4 to day 15 after the primary bleeding event. CT angiography (CTA) and perfusion scans were conducted on days 5, 10, and 15 after the aSAH. If pathological, a digital subtraction angiography (DSA) was performed. VS was considered to be severe in cases of a stenosis of > 50% and was treated with intermittent or continuous intraarterial nimodipine. Systemic oral or intravenous nimodipine was prophylactically administered to all patients, as recommended [2, 19]. Patients with evidence of VS were additionally treated with induced hypertension by i.v. fluids and noradrenaline. The CCT and DSA examinations were performed and assessed by a neuroradiologist in the Department of Neuroradiology. The findings were based on written reports.

Clinical data including age, gender, location of the aneurysm, treatment, World Federation of Neurosurgery (WFNS), Fisher, and extended Glasgow Outcome Score (eGOS) were collected from the patient charts by a neurosurgeon blinded to the patient outcomes and radiological findings. The location of a DCI, if present, was evaluated with regard to the location of the aneurysm. The aneurysm location was defined as ACA (A1, lateral A1/A2, A2 segments of the anterior cerebral artery), AcomA (anterior communicating artery), MCA (middle cerebral artery), ICA (internal carotid artery or posterior communicating artery, PcomA), BA (basilar artery), PCA (posterior cerebral artery), and VA/SCA/PICA (vertebral artery, superior cerebellar artery, or posterior inferior cerebellar artery). Vascular territories were defined as follows: MCA and/or ACA territory for ICA aneurysms, ACA territory for ACA or AcomA aneurysms, MCA territory for MCA aneurysms, and PCA and/or posterior fossa (brainstem, cerebellum) territory for BA and VA/PICA/SCA aneurysms. Vascular territories of ACA, MCA, PCA, and posterior fossa were evaluated for DCI and categorized into “ipsilateral” or “contralateral” in regard to the location of the aneurysm. For midline aneurysms (AcomA/BA), the right hemisphere was defined as ipsilateral, the left one as contralateral. The WFNS score was determined on admission or before administering sedatives and was dichotomized into good (grades I–III) or poor (grade IV+V) for further calculations. The Fisher score was obtained from the initial CCT scan and split into 3 groups (grade I+II, grade III, grade IV). The extended Glasgow outcome scale (eGOS) was assessed at discharge and on follow-up after 3 to 12 months and considered as favorable (eGOS 5-8) or unfavorable (eGOS 1-4). Patients who were lost to follow-up were assumed to have an unfavorable outcome. The neurological examinations were performed by a neurosurgeon in the Department of Neurosurgery.

### Statistics

Data acquisition and statistical analyses were performed with IBM® SPSS Statistics 21 (IBM Corporation, Armonk, NY, USA) and Microsoft® Excel 16.16.11 (Microsoft Corporation, Redmond, WA, USA). Due to the exploratory character of the study, no a priori case number calculation was performed. Metric variables were tested for a normal distribution using the Shapiro-Wilk test. Normally distributed data were compared by means of *T*-tests for dependent or independent variables, as appropriate. Nominally scaled data were analyzed with the Chi-square or Fisher’s exact test. *P*-values < 0.05 were considered as significant. The confidence interval was assumed to be 95%. Artwork was created with Microsoft® Excel 16.16.11.

### Statement of human and animal rights

All procedures performed involving human participants were in accordance with the ethical standards of the institutional and/or national research committee and with the 1964 Helsinki declaration and its later amendments or comparable ethical standards. For this type of study, formal patient consent is not required. Ethical approval for this study was obtained from the Ethics Commission at the Medical Faculty of the Eberhard Karls University Tuebingen (ID: 160/2019BO2).

## Results

### Patient characteristics

After analysis of 408 patients, the inclusion criteria were met by 357 patients who were included in the study; see Table 1. Among these, 247 (69.2%) individuals were women, and 110 (30.8%) were men. The mean patient age was 55.8 years (SD = 13.4). The clinical condition on admission was considered good (WFNS I–III) in 55.5% (*n*=198) and poor (WFNS IV–V) in 44.5% (*n*=159) of the patients. More than half of the patients presented with an aSAH Fisher grade IV (55.6%, *n*= 200), 30.4% (*n*=109) with a Fisher grade III, and 13.7% (*n*=49) showed bleeding with a Fisher grade I or II. A favorable outcome was achieved by half of all included patients at discharge (49.9%, *n*=178) and even more on follow-up (61.3%, *n*=219). A total of 22.1% (*n*=79) of patients were lost to follow-up and were assumed to have an unfavorable outcome. Median follow-up time was 4 months after discharge (Q1=3, Q3=5).

**Table 1:** Criteria and number of patients excluded from the study

Exclusion criterion	*N*
Deceased within 14 days	30
Territorial infarction not caused by DCI	12
Incomplete CT data	9
Total	51

### Locations of aneurysms

The most frequent location for a ruptured aneurysm was the MCA with 25.8% (*n*=92) of cases, followed by the ACA with 22.1% (*n*=79), the ICA with 21.3% (*n*=76), the AcomA with 20.2% (*n*=72), the BA with 3.9% (*n*=14), and VA/PICA/SCA aneurysms with 6.7% (*n*=24). Details about the aneurysm sites are listed in Table 2. Treatment of the aneurysm varied significantly (*p*<0.001) in response to the location. Whereas endovascular treatment was preferably performed in ICA (57.9%, *n*=44) and BA (100.0%, *n*=14), surgical treatment was preferred in MCA (95.7%, *n*=88) and VA/PICA/SCA aneurysms (58.3%, *n*=14). Both methods were used almost equally for AcomA (51.4%, *n*=37 endovascular) and ACA aneurysms (50.6%, *n*=40 endovascular). The treatment method had no significant influence on the outcome on discharge (*χ*^2^[1]=0.23, *p*=0.88) or on follow-up (*χ*^2^[1]=0.28, *p*=0.6). Among all aneurysms, 41.5% (*n*=148) were located on the right side, 34.2% (*n*=122) on the left side, and 24.4% (*n*=87) in the midline (AcomA or BA). There was evidence for VS on TCD and CTA or DSA in 52.4% (*n*=187) of patients. Intraarterial nimodipine was administered in 31.7% (*n*=113) due to severe VS.

**Table 2:** Distribution of aneurysm location among the entire patient population

Aneurysm location		*N*	**%**		*N*	**%**
Anterior circulation		319	89.4%			
	ACA	79	22.1%	A1	7	2.0%
				Pericallosal artery	14	3.9%
				A1/A2/Acom transition	58	16.2%
	AcomA	72	20.2%	AcomA	72	20.2%
	MCA	92	25.8%	MCA	35	9.8%
				MCA bifurcation	57	16.0%
	ICA	76	21.3%	ICA	64	17.9%
				PcomA	12	3.4%
Posterior circulation		38	10.6%			
	BA	14	3.9%	BA	3	0.8%
				Basilar tip	11	3.1%
	VA/PICA/SCA	24	6.7%	PICA	12	3.4%
				VA	7	2.0%
				SCA	5	1.4%
				PCA	0	0.0%
Total		357	100%		357	100%

### Delayed cerebral ischemia

DCI occurred in 17.9% (*n*=64) of all patients. A trend was observed for women suffering from a DCI slightly more often with 20.2% (*n*=50) vs. 12.7% (*n*=14) of men (*χ*^2^[1]=2.92, p=0.09). Age was not associated with the occurrence of a DCI (95% CI [−3.50, 3.79], *t*[355]=0.08, *p*=0.94). There was a significant association between the presence of severe VS and the occurrence of a DCI (*χ*^2^[1]=44.86, *p*<0.001). Of all patients presenting with a DCI, 87.3% (*n*=55) had evidence of VS.

**Fig. 1:**
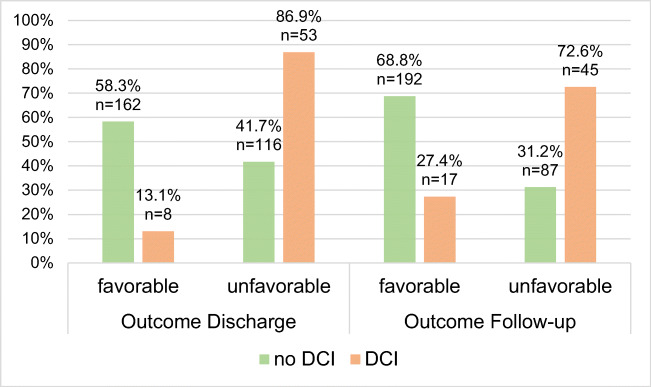
Distribution of favorable (=eGOS 1–4) and unfavorable (=eGOS 5–8) outcome at discharge and follow-up among aSAH patients with and without a DCI

DCI was significantly associated with a poor outcome on discharge (*χ*^2^[1]=42.95, p<0.001) and on follow-up (*χ*^2^[1]=39.78, *p*<0.001) since a favorable outcome was achieved by 12.7% (*n*=8) of patients with DCI vs. 58.2% (*n*=170) of patients with no DCI on discharge and by 26.6% (*n*=17) of patients with DCI vs. 68.9% (*n*=202) of patients with no DCI on follow-up (see Fig. 1). The occurrence of a DCI was significantly related with a worse WFNS score on admission (*χ*^2^[1]=16.20, *p*<0.001) as well as a higher Fisher grade (*χ*^2^[1]=6.14, *p*=0.046).

### DCI and aneurysm location

Aneurysm location had no effect on the incidence of delayed cerebral infarctions (*χ*^2^(1)=2.57, *p*=0.77). We also did not find a significant difference in the frequency of DCI occurring between anterior or posterior circulation aneurysms (18.2% vs 15.9%, *χ*^2^[1]=0.13, *p*=0.72); see Table 3.

**Table 3:** Frequency of DCI, multiple DCI, and no DCI according to the aneurysm location

Aneurysm location		DCI *N*	DCI %	Multiple DCI *N*	Multiple DCI %	No DCI *N*	No DCI %	Total *N*
Anterior circulation		58	18.2%	35	56.5%	261	81.8%	319
	ICA	15	19.7%	6	40.0%	61	80.3%	76
	ACA	14	17.7%	10	71.4%	65	82.3%	79
	AcomA	16	22.2%	14	82.4%	56	77.8%	72
	MCA	13	14.1%	5	31.2%	79	85.9%	92
Posterior circulation		6	15.8%	4	66.7%	32	84.2%	38
	BA	3	21.4%	3	100.0%	11	78.6%	14
	VA/PICA/SCA	3	12.5%	1	33.3%	21	87.5%	24
Total		64	17.9%	39	57.4%	293	83.1%	357

Percentages are shown by location

Among all patients who developed a DCI, the area most frequently affected was the ipsilateral MCA territory with 57.8% (*n*=37), followed by the ipsilateral ACA territory with 56.3% (*n*=36), the contralateral ACA territory with 46.9% (*n*=30), and the contralateral MCA territory with 37.5% (*n*=24). The PCA territory was affected less often with 14.3% (*n*=9) on the ipsilateral side and 4.8% (*n*=3) contralaterally. One patient showed a DCI of the ipsilateral (1.6%) and one of the contralateral (1.6%) posterior fossa territory.

#### DCI in the parent vessel’s territory

The area of the DCI was analyzed with regard to the location of the aneurysm. We found 75.0% (*n*=48) of all DCIs located in the respective vascular territory of the artery carrying the aneurysm. Patients with AcomA aneurysms showed a DCI in the right ACA territory in 93.8% (*n*=15) of cases, followed by ICA aneurysms that revealed a DCI in the ipsilateral ICA territory in 86.7% (*n*=13). In ACA aneurysms, an ipsilateral DCI of the ACA territory occurred in 64.3% (*n*=9), and in MCA aneurysms, an ipsilateral DCI of the MCA supply area was found in 69.2% (*n*=9) of cases. Posterior circulation aneurysms were associated with DCI in the same vascular territory in up to one-third of cases: 33.3% (*n*=1) in BA aneurysms and 0.0% (*n*=0) in VA/PICA/SCA aneurysms. A total of 81.0% (*n*=47) of patients with a DCI after rupture of an anterior circulation aneurysm but only 16.7% (*n*=1) of patients with a posterior circulation aneurysm suffered from a DCI in the vascular territory of the aneurysm’s parent vessel (Fisher’s exact, *p*=0.003).

#### DCI among all territories

**Fig. 2:**
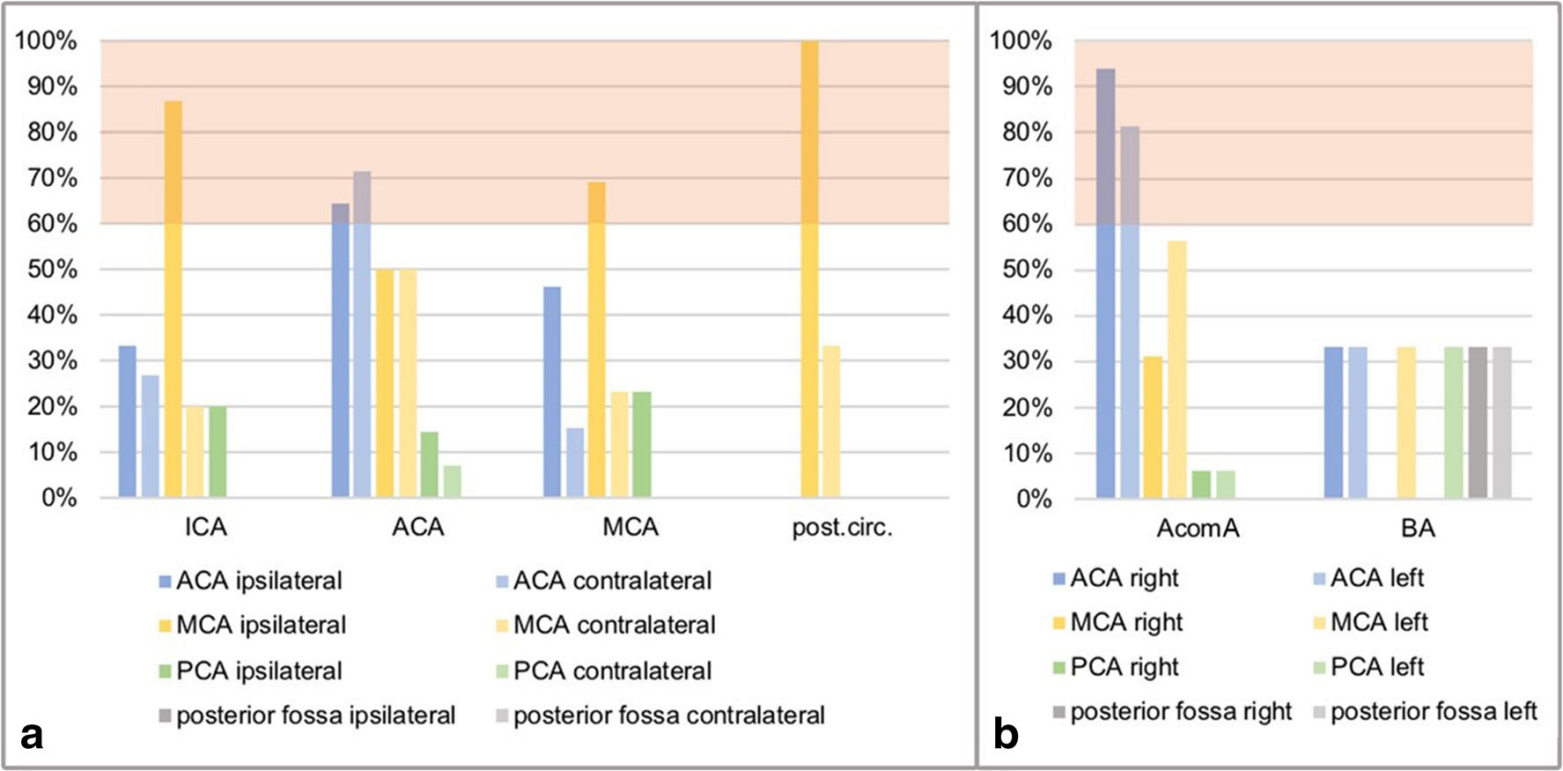
Frequency of different DCI territories according to the location of the aneurysm (*x*-axis, aneurysm location; *y*-axis, frequency within the location of vascular territories affected by the DCI; red area, territories affected > 60% of cases within the aneurysm location). **a** Lateralized aneurysms, **b** midline aneurysms

The distribution of DCIs according to the location of the aneurysm can be viewed in Fig. 2. For ICA aneurysms, the area most at risk for a DCI was the ipsilateral MCA territory (86.7%, *n*=13), followed by the ipsilateral ACA territory (33.3%, *n*=5). In ACA aneurysms, the contralateral ACA territory was affected slightly more often (71.4%, *n*=10) than the ipsilateral side (64.3%, *n*=9). Patients with AcomA aneurysms primarily presented with a right-sided DCI of the ACA territory (93.8%, *n*=15), followed by a left-sided ACA DCI (81.3%, *n*=13). In MCA aneurysms, the ipsilateral MCA territory was most frequently affected by the DCI (69.2%, *n*=9). However, almost half of the patients also presented with a DCI of the ipsilateral ACA (46.2%, *n*=6).

DCI in patients with BA aneurysms occurred equally in the right and the left ACA, the left MCA, the left PCA territory, and the right and the left posterior fossa (33.3%, *n*=1, respectively). We found that 100.0% (*n*=3) of patients with a VA/PICA/SCA aneurysm developed a DCI in the ipsilateral MCA territory, and 33.3% (*n*=1) also showed a DCI of the contralateral MCA territory.

A probability of more than 60% to develop a DCI in a specific vascular territory was reached for the ipsilateral MCA territory in ICA, MCA, and VA/PICA/SCA aneurysms and for both ACA territories in ACA and AcomA aneurysms (see Fig. 2). Either the ipsilateral ACA or MCA territory was affected in 100.0% of patients with AcomA or VA/PICA/SCA aneurysms, in 93.3% with ICA, 92.3% with MCA, and 78.6% with ACA aneurysms but only in 33.3% of patients with BA aneurysms.

Multiple territories were affected by a DCI in 60.9% of patients (*n*=39) and occurred in 100.0% of BA aneurysms, 87.5% of AcomA aneurysms, 71.4% of ACA aneurysms, 40.0% of ICA aneurysms, 38.5% of MCA aneurysms, and 33.3% of posterior circulation aneurysms other than BA; see Table 3. Multiple infarctions were associated with a significantly worse outcome on follow-up (*χ*^2^[1]=9.67, *p*=0.003) with 48.0% having a favorable outcome if only one territory was involved vs. 12.8% in cases of multiple infarctions, but not on discharge (Fisher’s exact test, *p*=0.36) with 16.7% vs. 10.3%, respectively.

## Discussion

The aim of this study was to identify the territory most at risk for the development of a DCI according to the location of the aneurysm since focal neuromonitoring methods are highly dependent on the probe’s location. Systematic investigations on this topic are rare. One study involving patients with severe VS with or without a DCI showed a good correlation when placing the focal probe into the ipsilateral territory of the aneurysm carrying vessel for ICA and MCA aneurysms but not for other locations. However, the calculations were made without distinguishing between the detection of VS and DCI [21]. Another work revealed a relation between the location of the infarctions and the parent vessel of the aneurysm in 79% of cases of single infarctions, but not in patients with multiple infarctions, which tended to occur more distantly [13]. There was no risk assessment with regard to the location of the aneurysm. Our data show a similar percentage of 75% of DCI occurring in the vascular territory of the artery carrying the aneurysm with an even higher number of 81% in anterior circulation aneurysms.

The presented patient population reflects the typical characteristics of patients with aSAH. The mean patient age was relatively young compared with other types of hemorrhagic or ischemic strokes, supporting data from the literature [6, 8]. As expected, there was a female predominance [10, 19].

An association between the development of a DCI and age has been postulated in previous studies [11] but was not found in our population. However, we observed a trend toward women being affected by DCI more frequently, supporting prior findings [5]. As expected [20], DCI showed a strong association with poor short- and long-term outcomes. In addition, DCI was associated with higher Fisher grade although this relationship has not primarily been described [3]. The modified Fisher scale has been shown stronger relations with VS and DCI [18] and was not investigated in this study.

The MCA territory was affected by a DCI most frequently in our cohort. On the one hand, his finding can be explained due to the high incidence of MCA aneurysms. On the other hand, ICA and posterior circulation aneurysms also most frequently presented with DCI in the MCA territory. The anatomical location of the MCA and its likely proximity to the subarachnoid blood clot in these aneurysms as well as the larger size of the MCA territory compared to the ACA and PCA territories are possible reasons for these findings.

The right hemisphere was defined as “ipsilateral” in midline aneurysms since intraparenchymal probes are often placed on the assumed non-dominant right side. This should serve as an easy to apply method for everyday clinical routine. Assessing the relation of lateralized blood load and the side of DCI might provide more detailed information for midline aneurysms, which was not the subject of this investigation.

Our data could identify the territory most at risk for DCI with a probability of > 60% for aneurysms in different locations, except for BA aneurysms, which showed DCI with equal frequency in 6 out of 8 possible vascular territories. In ACA and AcomA aneurysms, both ACA territories frequently showed infarctions; however, these patients tended to suffer from bilateral DCIs since more than 90% of patients with AcomA and over 70% of patients with ACA aneurysms (also) showed a DCI on the right (AcomA) or ipsilateral (ACA) side. This might be the result of the anatomical proximity of the two anterior cerebral arteries and the emerging blood clots after aSAH, since other lateralized aneurysms did not show bilateral DCI with a similarly high frequency. Focal neuromonitoring in the right (AcomA) or ipsilateral (ACA) ACA area therefore seems reasonable, and bilateral probes might not necessarily be required, although bilateral monitoring should be considered in high-risk patients [7] especially since TCD has been shown to detect VS less reliably in the ACA [1, 9].

Patients with ICA, MCA, or VA/PICA/SCA aneurysms revealed a DCI most probably and in more than 60% of cases in the ipsilateral MCA territory followed by the ACA territory in ICA and MCA aneurysms. In ACA, AcomA, ICA, MCA, and VA/PICA/SCA aneurysms, the ipsilateral ACA and the ipsilateral MCA territory were commonly affected by the DCI, both areas experiencing more than 90% of DCIs among AcomA, ICA, MCA, and VA/PICA/SCA aneurysms and almost 80% of DCIs among ACA aneurysms. Therefore, local intraparenchymal neuromonitoring in the ACA/MCA watershed area might detect the vast majority of DCIs for all aneurysm locations, except for BA aneurysms. We suggest the (additional) use of non-focal monitoring methods after BA aneurysm rupture, e.g., TCD or perfusion CT scans at a higher frequency.

DCI in the PCA territory as well as in the posterior fossa was rare and occurred less frequently than in the ACA and MCA territories. Patients with BA, AcomA, and ACA aneurysms were prone to suffer from multiple infarctions in 70–100% of cases and should be given special attention, whereas multiple infarctions occurred in less than 40% among the other aneurysm locations.

Some data have suggested lower rates of DCI after the rupture of a posterior circulation aneurysm [11], which could not be proven in other investigations [21]. Our data show the highest rates of DCI in AcomA and BA aneurysms and the lowest rates in VA/PICA/SCA aneurysms—a difference in the incidence of DCI between anterior and posterior circulation arteries could not be established.

## Conclusion

To our knowledge, this is the largest series of aSAH patients investigating the relationship between the location of the ruptured aneurysm and the DCI. Our data showed no difference in the frequency of DCI occurring in anterior or posterior circulation aneurysms. However, there were differences in the location of the DCI.

According to our findings, local intraparenchymal neuromonitoring in the ACA/MCA watershed area might detect about 80% of DCI after rupture of ACA aneurysms and more than 90% of DCI for all other aneurysm locations, except for BA aneurysms.

Patients with AcomA and ACA aneurysms frequently showed bilateral infarctions of the ACA territory with a high probability of detecting a DCI in the ipsilateral (or right-sided) ACA territory; therefore, bilateral focal probes might be considered in high-risk patients.

Only patients with BA aneurysms showed infarctions in variable areas, and they tended to develop multiple infarctions. We suggest that non-focal monitoring methods should be preferably used in these patients.

Our findings might be of value when deciding how and where to invasively monitor aSAH patients in accordance with the respective aneurysm site.

### Limitations of the study

This is a retrospective single-center study. The results were calculated by univariable analysis. The number of patients with a DCI after rupture of a posterior circulation aneurysm is limited. To confirm our findings especially in BA aneurysms, larger cohorts should be investigated. The proposed monitoring sites and methods should be further evaluated in prospective studies.

## Data Availability

The datasets generated during and/or analyzed during the current study are available from the corresponding author on reasonable request.
